# War in Ukraine as a mental health challenge of Czech health care workers

**DOI:** 10.1192/j.eurpsy.2024.234

**Published:** 2024-08-27

**Authors:** M. Janoušková, M. Kuklová, P. Čermáková, J. Pekara, J. Šeblová, D. Šeblová

**Affiliations:** ^1^Department of Epidemiology, Second Faculty of Medicine, Charles University; ^2^Department of Paramedic Science, Medical College, Prague; ^3^Emergency Department, Kladno Regional Hospital, Kladno, Czech Republic

## Abstract

**Introduction:**

Czech health care workers recently experienced serious challenges to their mental health. After the COVID-19 pandemic that was extremely stressful, a war in Ukraine caused a flood of refugees that needed health care. Although the Czech Republic does not have borders with Ukraine, it welcomed more than 400,000 refugees in 2022.

**Objectives:**

The aim of this study was to investigate the association between depression and working with Ukrainian refugees among health care workers and the nature of emotional burden connected with this situation.

**Methods:**

We use data from an online survey of the Czech COVID-19 HEalth caRe wOrkErS (HEROES) Study collected in September - November 2022 (n=1,076). We combined quantitative binary logistic regression and qualitative content analysis of answers to an open-ended question (“How does the current situation of war in Ukraine affect your mental well-being and working conditions?”). Logistic regression estimated odds ratio (OR) of at least moderate depression, defined as => 10 points on the Patient Health Questionnaire.

**Results:**

Among our participants (75.1% women, mean age 46 (SD 11.0)), 62.1% had experience of working with Ukrainian refugees, and 13.8% reported moderate to severe depression. Logistic regression model (adjusted for potential confounders) indicated that health care workers who worked with Ukrainian refugees had slightly greater chance of having depression, but the association was not statistically reliable (OR 1.05; 95% CI 0.59-1.86). Out of all survey respondents, 867 replied to an open-ended question. As follows from qualitative analysis, three categories of psychological strain were described by the health care workers: 1) specificity of work with the refugee patients (e.g. language barrier, increased workload, opinion conflicts), 2) insecurity, threat of war and fears about future (regarding global and nuclear war, security, future of kids, economic burden, etc.), 3) grief and compassion for the suffering of refugees. It was also frequently mentioned in the responses that war is a greater threat to health care workers than the COVID-19 pandemic.

**Conclusions:**

There is a slight association between working with refugees and depression. However, health care workers are also endangered by general fears of war and insecurity in a nearby country. In this changing world, it is of the greatest importance to pay attention to resilience building and stress prevention programs. Further, health care workers should be offered psychological support and practical resources to deal with the varying workload.

**Disclosure of Interest:**

None Declared

